# Molecular diagnosis of *Toxoplasma gondii *infection in cerebrospinal fluid from AIDS patients

**DOI:** 10.1186/1743-8454-6-2

**Published:** 2009-03-06

**Authors:** Yenisey Alfonso, Jorge Fraga, Carlos Fonseca, Narciso Jiménez, Taimy Pinillos, Alberto J Dorta-Contreras, Raymundo Cox, Virginia Capó, Olga Pomier, Francisco Bandera, Dora Ginorio

**Affiliations:** 1 Parasitology Department, Institute of Tropical Medicine 'Pedro Kourí', PO Box 601, Marianao 13, Ciudad de La Habana, Cuba; 2 Health Care Division, Institute of Tropical Medicine 'Pedro Kourí', PO Box 601, Marianao 13, Ciudad de La Habana, Cuba; 3 Central Laboratory of Cerebrospinal Fluid (LABCEL), PO Box 10049, Ciudad de la Habana, Cuba

## Abstract

**Background:**

Toxoplasmic encephalitis (TE) is one of the most common opportunistic infections in immunocompromised patients. In Cuba, despite the highly active antiretroviral therapy, TE is still the most important cause of cerebral mass lesions in patients infected with the human immunodeficiency virus (HIV). The detection of *Toxoplasma gondii *by PCR may facilitate the diagnosis and follow-up of TE in acquired immunodeficiency syndrome (AIDS) patients by direct identification of parasite DNA in clinical samples. The aim of the present study was to evaluate a rapid PCR method using the B1 gene to detect *T. gondii *in cerebrospinal fluid (CSF) samples from patients with suspected TE.

**Methods:**

CSF samples from AIDS and HIV-negative patients were analyzed. Patients were divided into two groups according to the Centre for Disease Control and Prevention (CDC) criteria for AIDS-related TE: AIDS patients with suspected neurotoxoplasmosis and AIDS and HIV-negative patients with other confirmed neurological diseases but no suspicions of TE. Predictive values, diagnostic accuracy, sensitivity and specificity of the PCR B1 method were calculated.

**Results:**

The results obtained from 190 patients showed that this assay has a good sensitivity and specificity (83.3% and 95.7%, respectively) for the diagnosis of TE in AIDS patients.

**Conclusion:**

PCR using the B1 gene and B22/B23 set of primers is a single, rapid and reliable method that may be valuable for discrimination between toxoplasmosis and other central nervous system (CNS) diseases.

## Background

Toxoplasmosis, caused by the protozoan *Toxoplasma gondii*, is a common parasitic infection in humans which has a cosmopolitan distribution. The prevalence of *T. gondii *infection varies among different geographical regions. The infection is characterized by non-specific symptoms with the consequent formation of cysts that may remain in latent form in many organs [1]. Reactivation of a latent infection occurs in immunocompromised patients causing life-threatening disease, especially encephalitis [2,3]. In our country, toxoplasmic encephalitis (TE) is still the most important cause of cerebral mass lesions in patients infected with human immunodeficiency virus (HIV) [4]. Therefore, a rapid diagnosis of TE is crucial for these patients with impaired immune functions, because early diagnosis and treatment may improve the clinical outcome. Although brain biopsy can establish a definitive diagnosis of TE it is an invasive and risky procedure associated with significant morbidity and mortality, while only half of the TE cases are confirmed [5].

It is standard practice to establish the presumptive diagnosis of the disease according to the Centre for Disease Control and Prevention (CDC) criteria for acquired immunodeficiency syndrome (AIDS)-related TE [6], but this is not infallible. For example, anti-*Toxoplasma *immunoglobulin detection may be unreliable in immunodeficient individuals, who fail to produce significant titers of specific antibodies [7]. The clinical presentation is indistinguishable from other neurologic disease frequently occurring among these patients [8]. The so-called "typical" lesions in the brain, detected by computed tomography (CT) or magnetic resonance imaging (MRI) are found in about 90% of the cases. However, these highly suggestive images of TE are not pathognomonic [9]. Upon the detection of an intracerebral suggestive lesion, an empirical treatment is usually initiated. In which case, the subsequent clinical and radiological improvement of the patient is considered a good criterion for diagnosis confirmation. Nevertheless, this approach may be used excessively in areas with high *T. gondii *seroprevalence [10].

Since the above mentioned criteria establish only a presumptive diagnosis, the need for less invasive, more sensitive, rapid and specific diagnostic methods is crucial for immunocompromised patients. For this reason, several studies for the diagnosis of TE employing PCR on cerebrospinal fluid (CSF) and blood samples have been reported [8,11-17]. As a result, several sets of primers for different DNA targets have been designed and each one of them tested on a small number of biological samples from different body sites [18], making a general consensus presently impossible [19]. Moreover, no assays have been sufficiently optimized and validated with a large number of well-characterized specimens [18]. Thus, the evaluation of each PCR in a large number of patients is extremely important for comparative laboratory studies, especially when the variability of conditions such as in the molecular diagnosis of toxoplasmosis is high.

The aim of this study was to evaluate a rapid PCR for TE diagnosis, using a set of primers for the most extensively used molecular target in a large number of CSF samples from immunocompromised patients.

## Methods

### Parasite preparation

*T. gondii *RH tachyzoites (2 × 10^5^/ml) were inoculated intraperitoneally into five OF1 Swiss mice. Tachyzoites were harvested three days later by peritoneal lavage collected in 5 ml of phosphate-buffered saline (PBS), and centrifuged at 1000 × *g *for 10 min. The pelleted parasites were resuspended and washed twice in PBS. Cells were counted in a Neubauer chamber and diluted in PBS to a concentration of 5 × 10^5 ^cells/ml.

### Patients

Approval for the study was obtained from the ethical committees of the Institute of Tropical Medicine "Pedro Kourí" and "Miguel Enriquez" Hospital using international criteria. Patients were informed about the study and written informed consent was obtained from each one of them or their closest relative. Clinical, immunological, radiological and laboratory data were collected from clinical charts.

The study included a total of 132 AIDS patients admitted to the clinical wards at the Institute of Tropical Medicine "Pedro Kourí", and 58 samples of HIV-negative patients from "Miguel Enriquez" Hospital, both in Havana, Cuba. Neurological symptoms were present in all patients. Before specific therapy was started, a spinal tap was performed, to collect 1 ml of CSF from each patient, and samples were stored at -20°C until analyzed.

Patients were divided into two groups. Group I (n = 48) consisted of AIDS patients with suspected neurotoxoplasmosis according to the CDC criteria for AIDS-related TE [6] which includes the following clinical and radiological features: I) recent onset of a consistent focal neurological abnormality with intracranial disease or reduced level of consciousness; II) a lesion having a mass effect evidenced by CT imaging and III) serum antibody to *T. gondii *or successful response to treatment of toxoplasmosis. Group II (n = 142) consisted of 84 patients with other AIDS-related disorders (25 neurocryptococcosis, 11 tuberculosis, 6 non-Hodgkin lymphoma, 9 viral meningitis, 12 HIV encephalitis, 21 active febrile illness not suspected of being toxoplasmosis) and 58 HIV-negative patients with inflammatory or non-inflammatory neurological disease.

### DNA extraction

For the DNA to be used as positive control for PCR, the phenol-chloroform-isoamyl alcohol (25:24:1) extraction method was employed [20] on a previously-counted pellet of RH strain *T. gondii *cells. All CSF samples were individually concentrated by centrifugation at 3000 × *g *for 10 min. Sediments were re-suspended in 40 μl of lysis buffer (10 mM Tris-HCl pH 8.3, 1.5 mM MgCl_2_, 50 mM KCl, 0.1 mg of gelatin per ml, 0.5% Tween 20, 20 μg of proteinase K (Promega, Madison, USA) and incubated at 55°C, with shaking for 90 min. After inactivating the proteinase K at 94°C for 10 min, the tubes were centrifuged at 10,000 × *g *for 5 min, and the supernatants collected [21,22].

### PCR analytical sensitivity and specificity

To determine the PCR sensitivity, two-fold serial dilutions ranging from 5 × 10^5 ^to 1 cell of the parasite (RH strain) were prepared and DNA was extracted as previously described [21,22]. The DNA obtained was used for PCR under the same conditions as for clinical samples. Specificity was determined by PCR amplification of DNA extracted from yeasts (*Cryptoccocus neoformans, Candida albicans, Candida parapsilopsis*), gram positive cocci *(Staphylococcus aureus, Staphylococcus epidermidis, Streptoccocus pneumoniae*), gram negative rods *(Serratia marcescens, Pseudomona aeruginos, Escherichia coli, Haemophilus influenzae*), gram negative coccus (*Neisseria meningitides*) and herpes virus (*Herpes simplex, Epstein Barr, Varicela Zoster, Cytomegalovirus*).

### Detection of *T. gondii *DNA by PCR

From the CSF samples, *T. gondii *DNA was detected by the amplification of a fragment of 115-bp of B1 gene [23], using primers B22 and B23 as previously described by Bretagne *et al*. [24]. The reaction conditions were tested using a large number of modifications to optimize the PCR conditions: temperature for primer annealing (58°C, 59°C, 60°C), MgCl_2 _concentration (1.5, 2.0, 3.0, 4 mM), Taq DNA polymerase concentration (0.5, 1.0, 1.5, 2.0, 3.0 U) and primer concentration (0.05, 0.1, 0.2, 0.3, 0.4 μM). Consequently the proportion of DNA from CSF samples in the mixture reaction was also optimized. The optimized amplification was performed in 25 μl of reaction mixture containing 10 mM Tris-HCl, pH 8.5, 50 mM KCl, 1.5 mM MgCl_2_, 0.2 μM of each primer (B22 and B23), 200 μM of each deoxynucleoside triphosphate and 1.5 U Taq DNA polymerase (Promega). Ten microliters of extracted DNA from CSF samples were added as template. The PCR conditions were 94°C for 5 min, followed by 35 cycles of 94°C for 30 s, 59°C for 30 s, and 72°C for 30 s and a last extension step at 72°C for 10 min.

Each amplification assay contained two negative controls (ultrapure water and a negative control for DNA extraction) and one positive control (DNA extracted from RH *T. gondii *tachyzoites). Physical separation for mixture preparation, DNA extraction and visualization of PCR products were carried out and decontamination procedures were used in all areas to avoid contamination by amplicons. The PCR products were analyzed by 2% agarose gel electrophoresis in tris-borate-EDTA (TBE) buffer and stained with ethidium bromide (0.5 mg/ml). The DNA fragments were visualized under UV illumination.

Clinical samples with positive PCR results were tested twice. For the negative samples, an inhibition test was performed to discard the presence of inhibitors. (i.e., DNA equivalent to 5 cells of *T. gondii *RH was added to the negative DNA sample).

### Statistical analysis

Predictive values, diagnostic accuracy, sensitivity and specificity of the PCR B1 method were calculated using a 2 × 2 contingency table and recognized formulae [25-27] in which the CDC criteria for TE were used as a "gold standard".

## Results

**Figure 1:**
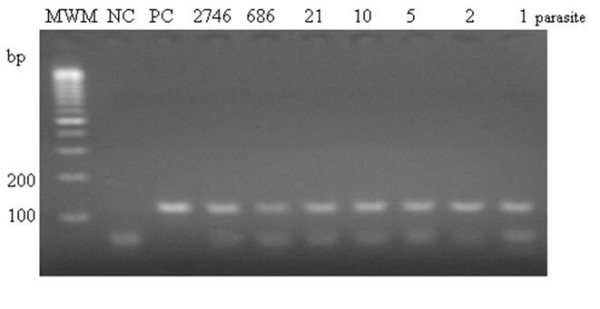
**Agarose gel electrophoresis analysis of PCR amplifications products performed with different numbers of tachyzoites**. Lane MWM: Molecular Weight Marker 50 bp (Amersham, USA). Lane NC: Negative Control. Lane PC: Positive control.

To determine the usefulness of the PCR for the diagnosis of TE in immunocompromised patients, 190 CSF samples from AIDS and HIV-negative patients were studied. After optimization of PCR conditions (data not shown), the assay was sensitive enough to detect up to one tachyzoite of *T. gondii*. A representative agarose gel of PCR products is shown in Figure 1. In order to investigate the analytical specificity of PCR, DNA from a variety of microorganisms that could potentially be present in the CSF of AIDS patients was used as target for the PCR reaction. No amplification product was observed when DNA from these specimens was present in the mixture reaction, thus demonstrating the absolute specificity of the set of PCR primers used (data not shown).

PCR diagnostic sensitivity and specificity were evaluated using the CDC criteria as the gold standard for AIDS-related TE to detect *T. gondii *central nervous system (CNS) infection in patients, with and without suspected infection. The PCR results are shown in Table 1. In group I, 40/48 patients with suspected TE had PCR positive results. In group II, corresponding to patients without suspected TE, six samples produced positive results while the remaining 136 (95.7%) resulted negative. None of these positive samples were from HIV-negative patients.

**Table 1:** Relation between the PCR results from CSF samples and the presence or absence of toxoplasmic encephalitis (TE).

**PCR results**	**Patients with TE (I)**	**Patients without TE (II)**	**Total No. of samples**
**Positive PCR**	40(83.3%)^1^	6	46

**Negative PCR**	8	136 (95.7%)^2^	144

**Total No. of samples**	48	142	190

^1 ^Diagnostic sensitivity

^2 ^Diagnostic specificity

The data represent a diagnostic sensitivity of 83.3% and a 95.7% of specificity. The positive predictive value of the assay was 86.9% while negative predictive value was 94.4%. The diagnostic accuracy was 92.6%, indicating the assay is reliable.

## Discussion

The introduction of Highly Active Antiretroviral Therapy (HAART) has resulted in the decline of the incidence of opportunistic infections in the CNS in AIDS patients [5]. Particularly in Cuba, despite the universal use of HAART, TE is still the most important cause of cerebral mass lesions among HIV-infected patients [4]. Although a definitive diagnosis of the disease relies on the identification of parasites by histopathology, the clinical and radiological data could be complemented by a less invasive approach [17] that confirms the presence of parasites in body fluids, resulting in a better clinical management of the disease. The selection of the primers is also an essential step for successful PCR. Chabbert *et al*. [28] previously reported the high sensitivity of the B22 and B23 set of primers when compared with other *T. gondii *sequence primers. Due to their higher sensitivity, specificity and practicability, the use of these primers was recommended for the detection of *T. gondii *DNA in immunocompromised patients where Brazilian researchers found they gave a good diagnostic sensitivity [16,17]. The B1 gene has been the most widely used target for the diagnosis of toxoplasmosis [19]. Hence, it was expected that the B1 PCR employed here would prove to be useful for the detection of *T. gondii *CNS infection. The evaluation of the large cohort used in this study adds weight to this hypothesis.

The results of the analytical sensitivity assay (1 parasite) was similar to previous studies that used sets of primers for B1 gene and was able to detect either a single organism from a crude cell lysate [23,29] or 10 tachyzoites in presence of 100,000 human leukocytes [23]. These results show that very small numbers of *T. gondii *can be detected. The diagnostic sensitivity found in this study was 83.3% (Table 1). This means that 40 patients out of 48 with suspected neurotoxoplasmosis gave positive results for the presence of *T. gondii*, demonstrating that PCR is a diagnostic technique with relatively high sensitivity for TE in CSF samples. Eight AIDS patients with suspected TE had negative results and in one of them the necropsy confirmed the diagnosis of TE. None of these patients received treatment before the spinal tap. Therapy is an important element for the sensitivity of PCR, since previous studies reported that anti-*Toxoplasma *therapy decreases diagnostic sensitivity, especially when the samples are collected after the first week of treatment [30].

The sensitivities previously reported for TE diagnosis from CSF range widely in value (17% – 100%) with an average of 59% (97/164 patients with TE from 17 studies) [19]. The sensitivity obtained in our work corresponds to the range reported for CSF and was superior to several studies that use CSF and nested reactions for B1 gene [12,31]. Also, it is noteworthy that the sensitivity of a PCR depends on many factors such as physicochemical conditions of the reaction, the concentration and nature of DNA target, the selected PCR primers and the nucleic acid extraction method [32]. In our case, optimization of the PCR conditions was carefully carried out enhancing the analytical sensitivity and specificity of the assay. An additional advantage is the frequency in which the B1 gene is present in the parasite genome [33]. Is well known that the number of copies of the DNA target in the genome of interest is an essential factor for the assay sensitivity and also that the use of the same primers does not necessarily ensure identical results for different laboratories [32]. In relation to this, a study carried out in Brazil by Vidal *et al*. [16] used CSF samples from immunocompromised patients and the same set of primers for the B1 gene as used here. They reported 100% sensitivity while our sensitivity for the present study was lower, even though similar PCR conditions were used. On the other hand the above mentioned study included 12 patients, while we used samples from four times more patients with TE. Nevertheless, the DNA extraction methods differed between the laboratories and this fact may play an important role in the assay performances and significantly influence the assay sensitivity. In our experience, as previously reported, the DNA extraction protocol was chosen after comparing four different extraction methods [22]. In comparison with other in-house DNA extraction methods the protocol here used is rapid, reproducible, and simple and it could be recommended for routine PCR diagnosis of TE in places with limited resources.

The laboratory conditions are crucial to raise the sensitivity and specificity of the PCR technique, especially when it is used on clinical specimens. For this reason, an inhibition test was carried out for every sample producing negative results in order to rule out the presence of PCR inhibitors. Moreover, negative controls were used to check for contamination and all the necessary cautions were taken in order to prevent possible risks of contamination that frequently happens in this kind of reaction.

As for the dignostic specificity, all six sample from AIDS patients without suspected TE with positive results, corresponded to patients presenting with other AIDS related diseases but without clinical or radiological evidence of cerebral toxoplasmosis upon admission. Although specificity of PCR in the diagnosis of TE has been extremely high in most of reports, false positive results have been described [16,17,34,35], particularly among immunocompromised patients, for whom it has been proposed that parasitemia may exist prior to, or during the course of cerebral toxoplasmosis [18]. In our case, clinical surveillance of these six patients was not made and the possibility of subsequent TE could not be excluded, making the results inconclusive. It is possible that the sporadic breakdown of tissue cysts may provide enough DNA in the CSF for parasite detection, in contrast to the large amount of parasite DNA that is found in evident TE [34]. These six individuals could have still some immunological capacity to destroy parasites released from the sporadic breakdown of cysts, and so prevent the reactivation of TE.

## Conclusion

PCR using the B22 and B23 primer sets for the diagnosis of TE in AIDS patients is a rapid, simple, reliable, sensitive and specific method, and can be used as a practical and alternative tool for the diagnosis of TE in immunocompromised patients. The method is easy to perform and less prone to contamination compared with nested PCR reaction and will help patients to gain the benefit of early anti-*Toxoplasma *treatment.

## Competing interests

The authors declare that they have no competing interests.

## Authors' contributions

JFN designed the study, performed coordination and drafted the manuscript. YA carried out the molecular studies and drafted the manuscript. CF, NJ, OP, FB, TP and AJDC participated in the design, data collection and review the clinical profiles of patients and the patients grouped. RC carried out the immunoassays. VC carried out the pathology studies and contributed to the manuscript. DG participated in the laboratory coordination and contributed to the manuscript. All authors read and approved the final version of the manuscript.
